# Impaired p53-Mediated DNA Damage Response Contributes to Microcephaly in Nijmegen Breakage Syndrome Patient-Derived Cerebral Organoids

**DOI:** 10.3390/cells11050802

**Published:** 2022-02-25

**Authors:** Soraia Martins, Lars Erichsen, Angeliki Datsi, Wasco Wruck, Wolfgang Goering, Eleftheria Chatzantonaki, Vanessa Cristina Meira de Amorim, Andrea Rossi, Krystyna H. Chrzanowska, James Adjaye

**Affiliations:** 1 Institute for Stem Cell Research and Regenerative Medicine, Medical Faculty, Heinrich-Heine University, 40225 Düsseldorf, Germany; soraia.martins@iuf-duesseldorf.de (S.M.); lars.erichsen@med.uni-duesseldorf.de (L.E.); wasco.wruck@med.uni-duesseldorf.de (W.W.); eleftheria.xtz@gmail.com (E.C.); vanessacristina.meiradeamorim@med.uni-duesseldorf.de (V.C.M.d.A.); 2 Institute for Transplantation Diagnostics and Cell Therapeutics, Heinrich-Heine University, 40225 Düsseldorf, Germany; angeliki.datsi@med.uni-duesseldorf.de; 3 Institute for Pathology, Medical Faculty, Heinrich-Heine University, 40225 Düsseldorf, Germany; wolfgang.goering@med.uni-duesseldorf.de; 4 IUF-Leibniz Research Institute for Environmental Medicine, 40225 Düsseldorf, Germany; andrea.rossi@iuf-duesseldorf.de; 5 Department of Medical Genetics, Children’s Memorial Health Institute, 04-730 Warsaw, Poland; k.chrzanowska@czd.pl

**Keywords:** iPSCs, cerebral organoids, disease modeling, p53, Neuronatin, NBS, microcephaly

## Abstract

Nijmegen Breakage Syndrome (NBS) is a rare autosomal recessive genetic disorder caused by mutations within nibrin (*NBN*), a DNA damage repair protein. Hallmarks of NBS include chromosomal instability and clinical manifestations such as growth retardation, immunodeficiency, and progressive microcephaly. We employed induced pluripotent stem cell-derived cerebral organoids from two NBS patients to study the etiology of microcephaly. We show that NBS organoids carrying the homozygous 657del5 *NBN* mutation are significantly smaller with disrupted cyto-architecture. The organoids exhibit premature differentiation, and Neuronatin (NNAT) over-expression. Furthermore, pathways related to DNA damage response and cell cycle are differentially regulated compared to controls. After exposure to bleomycin, NBS organoids undergo delayed p53-mediated DNA damage response and aberrant trans-synaptic signaling, which ultimately leads to neuronal apoptosis. Our data provide insights into how mutations within *NBN* alters neurogenesis in NBS patients, thus providing a proof of concept that cerebral organoids are a valuable tool for studying DNA damage-related disorders.

## 1. Introduction

Development of the nervous system is a strictly regulated process whereby neural progenitor cells (NPCs) rapidly proliferate, differentiate, and undergo oxidative stress. Neurons, being post-mitotic cells, display high rates of metabolism and mitochondria activity also contributing to a stressful environment which increases the susceptibility to DNA damage. Deficiency in DNA damage response (DDR) causes many syndromes with pronounced neuropathology [1,2]. Hypomorphic mutations within *Nibrin* (*NBN*), which encodes a DDR protein, leads to Nijmegen Breakage Syndrome (NBS)—a rare autosomal recessive genetic disorder belonging to the chromosomal instability syndromes [3].

Clinically, NBS is characterized by severe and progressive microcephaly, growth retardation, typical facial appearance, premature ovarian failure, deterioration of cognitive functions, immunodeficiency, chromosomal instability, and elevated sensitivity to ionizing radiation. In addition to microcephaly, other developmental abnormalities of the brain have been observed in a few patients, including neuronal migration disorder, agnesis of the corpus callosum and arachnoid cysts [4,5,6]. By the age of 20, more than 40% of NBS patients develop malignancy-associated diseases, predominantly of hematologic origin, which in addition to the recurrent infections are the major causes of death in these patients [7]. The worldwide prevalence is estimated at 1:100,000 live births; however NBS is particularly common in Eastern Europe with carrier frequencies as high as 1:155 in some populations of the Czech Republic, Poland, and Ukraine [8]. More than 90% of NBS patients are homozygous for a founder mutation—a five base pair deletion in exon 6 (657del5) within *NBN*. Due to alternative translation from a cryptic start site upstream of the deletion, this mutation leads to the truncation of the wild-type protein into two distinct fragments: a 26 kDa amino-terminal protein (p26) and a 70 kDa carboxy-terminal (p70), which retain some residual functions [9].

NBN together with MRE11 and RAD50 form the MRN complex which plays a central role in DNA damage signaling and repair, telomere maintenance, proper centromere duplication, cell cycle checkpoint activation and processing of stalled replication forks [10]. With NBN playing a multi-functional central role, it is not surprising that lymphoblasts and fibroblasts from NBS patients exhibit chromosomal instability with impaired cell cycle and regulation of apoptosis [11,12,13].

The functional consequences of *NBN* 647del5 during neurodevelopment remains largely unexplored. To this end, several studies based on *Nbn* conditional knockouts and inducible *Nbn*-deletion mice have been generated. These studies identified proliferation arrest and increased apoptosis in the neural progenitor cells due to over-activation of p53, which ultimately leads to microcephaly [14,15,16,17,18]. Although these studies have provided helpful information, it is necessary to compare and bridge the gap between these mouse models and the human phenotype of NBS.

Induced pluripotent stem cells (iPSCs) provide the best platform to derive a reliable human disease model to study the effects of *NBN* mutations in neurons derived from NBS patients. In fact, we previously reported that fibroblasts from NBS patients can be reprogrammed into iPSCs and thus by-passing premature senescence [19]. Transcriptome analysis of NBS fibroblasts and NBS-iPSCs unveiled de-regulated cancer related pathways such as p53, cell cycle and glycolysis [20]. Differentiation of the NBS-iPSCs into NPCs showed these cells have de-regulated expression of neural developmental genes in part due to the inability of NBS-NPCs to maintain normal levels of p53 [21].

Recent advances in in vitro culture of 3D cerebral organoids derived from iPSCs have illuminated the early mechanisms of mammalian neurodevelopment. Here, we took advantage of 3D cerebral organoids to analyze the underlying molecular mechanisms associated with microcephaly and other brain abnormalities seen in NBS patients. Compared to healthy controls, NBS organoids carrying the homozygous *NBN* 657del5 mutation are significantly smaller in size and have disrupted cyto-architecture. Both patient-derived organoids exhibit premature differentiation, additionally key pathways related to DNA damage response and cell cycle are differentially regulated compared to healthy controls. After exposure to bleomycin- an inducer of DNA damage, NBS organoids present a delay in the activation of cell cycle arrest due to their inability to stabilize p53.

Our data highlights insights into how hypomorphic mutations within *NBN* alters neurogenesis in NBS patients, thus providing a proof of concept that iPSC-derived cerebral organoids are a valuable tool which enable research into DNA damage-related disorders such as Nijmegen Breakage Syndrome.

## 2. Materials and Methods

### 2.1. iPSCs Derivation and Culture Methods

The iPSC lines derived from NBS patients (NBS1 and NBS8) as well as the control individuals CTR1 and CTR2 used in this study have been previously described [19,22,23,24]. One clone per iPSC line was used for further investigation. hiPSC lines were cultured in mTESR medium (StemCell Technologies, Vancouver, Canada) supplemented with Penicillin/Streptomycin (P/S) on Matrigel-coated plates (Corning, New York, NY, USA). The medium was changed every day and cells were passaged every 5–6 days using PBS without Calcium and Magnesium (Life Technologies, Waltham, MA, USA).

### 2.2. Generation of Cerebral Organoids

For the generation of cerebral organoids, iPSCs were differentiated as described [25], but with further optimization. On day 0, iPSCs were dissociated using TrypLE Express (Gibco, Waltham, MA, USA) and plated into a 96-well ultra-low-attachment (10,000 cells/well, Nucleon^TM^ Sphera^TM^, Thermo Scientific) in mTEST supplemented with 10 µM ROCK inhibitor Y-27632 (Tocris Bioscience, Bristol, UK). Neural induction was initiated on day 1 by adding Neural induction medium (NiM) (DMEM/F12, 20% Knock-out serum replacement, 1%NEAA, 0.5% GlutaMAX and 0.1mM 2-Mercaptoethanol (all from Gibco) with 10 µM SB-431542 (Tocris Bioscience), 5 µM Dorsomorphine (Tocris Bioscience) and 10 µM ROCK inhibitor Y-27632. The medium was changed daily. After five days, the spheroids were transferred to Neural differentiation medium (NdM) (Neurobasal, 2% B27, 1% GlutaMAX, 1% P/S (all from Gibco)) supplemented with 20 ng/mL of EGF and FGF2 (both PrepoTech, Cranbury, NJ, USA) in non-adhesive 100 mm dishes. Organoids were further cultured under continuous agitation (60 rpm) in a shaking incubator (New Brunswick S41i, Eppendorf) with daily medium change until day 16, and from day 16 onwards medium was changed every other day. At day 25, medium was replaced to NdM supplemented with 20 ng/mL of BDNF with medium change every 2–3 days. From day 40 onwards, organoids were kept in NdM with medium change every 4 days. To induce DNA damage, cerebral organoids at day 37 were incubated with 30 µg/mL of Bleomycin (Millipore, Darmstadt, Germany) for 72 h before harvesting for RNA extraction and for protein lysate preparation.

### 2.3. Single Cell Dissociation and 2D Neuronal Culture

Forty-day CTR1, NBS1 and NBS8 organoids were dissociated to a single cell suspension for 30 min using the Papain dissociation kit (Worthington, OH, USA) according to the manufactures’ instructions. 300,000 cells were replated into poly-L-ornithine and laminin (Sigma, Taufkirchen, Germany) coated coverslips in neural differentiation medium (Neurobasal, 1% NEAA, 1% N2, 1% P/S (all Thermo Fisher Scientific) supplemented with 1 µM of cAMP (Thermo Fisher Scientific, Rockford, IL, USA) and 10 ng/mL of BDNF, GDNF and IGF-1 (all Immuno Tools, Friesoythe, Germany).

### 2.4. Organoid Sectioning and Immunostainings

Cerebral organoids were fixed in 4% paraformaldehyde (PFA) for 30 min at room temperature, washed with PBS and dehydrated in 30% sucrose in PBS overnight at 4 °C. Subsequently organoids were transferred into embedding medium (Tissue-Tek OCT Compound 4583, Sakura Finetek, Umkirch, Germany), snap-frozen on dry ice and stored at −80 °C. Organoids were cut into 10 µm sections and captured in Superfrost Plus slides (Thermo Scientific) using a Cryostat Leica CM3050 S. Cryosections were permeabilized with 0.1% Triton X-100 for 10 min and blocked with 3% BSA in PBS for 1 h. Samples were then incubated with the following primary antibodies overnight at 4 °C: mouse anti-βIII-tubulin (1:200, CST #TU-20), mouse anti-SOX2 (1:100, Invitrogen, Thermo Fisher Scientific #20G5), rabbit anti-SOX2 (1:200, CST #3579S), guinea pig anti-DCX (1:200, SySy #326004), mouse anti-KI67 (1:200, CST #9449), rabbit anti-cleaved CASP3 (1:200, CST #9664). After washing with PBS, cells were then incubated with the appropriate secondary antibody conjugated with Alexa-488, Alexa-555 or Alexa-647 (1:500, Invitrogene, Thermo Fisher Scientific) for 1 h at RT. The nuclear stain Hoechst 33,258 (2 ug/mL, Sigma) was added at the time of the secondary antibody incubation. Slices were mounted with Fluoromount-G (SouthernBiotech, Bermingham, AL, USA) and fluorescent and confocal images were obtained using a LSM 700 microscope (Carl Zeiss, Jena, Germany), and processed using ZEN software (Carl Zeiss).

### 2.5. Quantitative Assessment of Cerebral Organoids and Image Analysis of Histological Sections

The size of the organoids was measured employing a Digital Microscope Leica DMS100. Two perpendicular measurements of the organoids diameter (µM) was determined using ImageJ and the mean was calculated. For quantification of the number of ventricular zones-like structures per organoid, organoid slides were stained with SOX2, DCX antibodies and nuclei stained with Hoeschst. For quantification of the thickness of the ventricular zones-like structure, the average of two measurements (µM) (0 and 45 degrees) starting from the apical membrane until the basal plate were performed (Figure 2H—solid line). The length of the apical membrane was measured as exemplified in Figure 2H by the dashed-line. To determine the number was SOX2^+^, KI67^+,^ DCX^+^, and βIII-Tubulin+ cells relative to the total number of cells, the integrated density for each antibody was calculated and divided by the integrated density of the nuclei staining. At least four sections per organoid were analyzed. Loop diameter, apical membrane length, and integrity intensity were measured using ImageJ software.

### 2.6. Reverse Transcriptase PCR (RT-PCR)

Total RNA was extracted from day 20 (three pooled organoids) or day 40 (single organoids) using TRIzol (Life technologies) and Direct-zol RNA Mini Prep (Zymo Research, Freiburg, Germnay) according to the manufacturer’s protocol. 500 ng of purified RNA was used for cDNA synthesis using TaqMan reverse transcription reagent (Applied Biosystems, Waltham, MA, EUA). cDNA was used for subsequent PCR analysis. To detect the presence of transcripts, RT-PCR reactions were performed using 20 ng of cDNA of CTR2 and NBS8 organoids using GoTaq^®^ NA Polymerase (Promega). Total RNA from commercially human fetal and adult brain (BioChain^®^) was used as positive control.

Transcripts abundance was determined by reverse transcription quantitative PCR (qRT-PCR) using the SYBR^®^ Green RT-PCR assay (Applied Biosystems). Amplification, detection of specific gene products, and quantitative analysis were performed using a ‘ViiA7’ sequence detection system (Applied Biosystem). The expression levels were normalized relative to the expression of the housekeeping gene *RPS16* using the comparative Ct-method 2^−∆∆Ct^. Experiments were carried out in biological triplicates except for 40 d organoids (technical triplicates). RT-qPCR data are depicted as mean values with 95% confidence interval. Primers are listed in Supplementary Table S5.

### 2.7. Microarray Analysis

100 ng of total RNA from three-pooled organoids arbitrarily selected from each genotype (CTR, CTR2, NBS1 and NBS8) at 20D and two arbitrarily selected single organoids from CTR2 and NBS8 at 40D was subjected to hybridization on the Human Clarion S Array (Affymetrix, Thermo Fisher Scientific) at the BMFZ (Biomedizinisches Forschungszentrum) core facility of the Heinrich-Heine Universität, Düsseldorf. RNA integrity was evaluated using a Fragment Analyzer (Advanced Analytical Technologies, AATI). Data analysis of the Affymetrix raw data was performed in the R/Bioconductor [26] environment using the package oligo [27]. The obtained data were background-corrected and normalized by employing the Robust Multi-array Average (RMA) method from the package oligo. Hierarchical clustering dendrograms and heatmaps were generated using the heatmap.2 function from the gplots package with the Pearson correlation as similarity measure and color scaling per gene [28]. Expressed genes were compared in Venn diagrams employing package VennDiagram [29]. Gene expression was assessed with a threshold of 0.05 for the detection *p* value which was calculated as described in Graffmann et al. [30]. Comprehensive functional analysis of the clustered GO biological processes and pathways (KEGG pathways, Reactome Gene Sets, Canonical pathways and CORUM) of the candidate genes was performed using Metascape tool (http://metascape.org, accessed on 8 August 2020) [31]. The default parameters were used: terms with *p* < 0.01, minimum overlap 3 and enrichment factor >1.5. To identify enriched brain regions in the NBS organoids, genes associated with fetal brain regions were downloaded from the Allen Brain Atlas (ABA) human developmental Brain NGS data (https://www.brainspan.org/, accessed on 8 May 2020) [32]. Brain regions are abbreviated by the acronyms: occipital neocortex—Ocx, primary motor-sensory cortex (samples)—M1C-S1C, amygdaloid complex—AMY, medial ganglionic eminence—MGE, posterior (caudal) superior temporal cortex (area 22c)—STC, upper (rostral) rhombic lip—URL, caudal ganglionic eminence—CGE, dorsal thalamus—DTH, anterior (rostral) cingulate (medial prefrontal) cortex—MFC, dorsolateral prefrontal cortex—DFC, orbital frontal cortex—OFC, lateral ganglionic eminence—LGE, inferolateral temporal cortex (area TEv, area 20)—ITC, hippocampus (hippocampal formation)—HIP, ventrolateral prefrontal cortex—VFC, parietal neocortex—PCx.

Normalized and log2-transformed transcriptome data filtered for a high coefficient of variation (cv > 75% quantile) were subjected to gene set variation analysis (GSVA) [33]. GSVA calculates enrichment scores for each sample and each gene set. We then calculated the differential expression at the pathway level between the 40-day and 20-day samples via the tests from the Bioconductor limma package [34]. Resulting *p*-values were corrected for multiple testing via the qvalue method [35]. GSVA enrichment scores with a q-value less than 0.05 were used for hierarchical clustering and heatmap generation with the heatmap.2 function from the gplots package [28].

### 2.8. Western Blot

Cells of five pooled organoids were washed with PBS and then lysed in RIPA buffer containing complete protease and phosphatase inhibitor cocktail (Roche). Lysates were cleared by centrifugation at 20,000× *g* for 10 min and quantified with the Pierce™ BCA Protein Assay kit (Thermo Scientific). 25 µg of the lysates were then separated on NuPAGE 4–12% Bis-Tris gels (Invitrogen) and blotted to a 0.45 µm nitrocellulose membrane for 3 h at 300 mA. The blots were blocked in PBS/0.05%Tween20 containing 5% skim milk and then probed with the following primary antibodies over night at 4 °C: mouse anti-P53 (1:100), Merck rabbit anti-H2AX (1:1000, Cell Signaling), mouse anti β-actin (1:4000, Cell Signaling). After washing the blots three times with PBS/0.05%Tween20 they were incubated with the appropriate secondary antibody: goat anti-mouse IRDye 680RD and 800CW as well as goat anti-rabbit IRDye 680RD and 800CW (all from LI-COR Biosciences, Lincoln, NE, USA). Following three times washing with TBS/0.05% Tween20, the fluorescent signals were quantified by applying the Odyssey infrared imaging system (LI-COR Biosciences).

### 2.9. Flow Cytometry

The cell marker expression of the 40-day organoids was analyzed by Flow cytometry analysis. To conduct this analysis, 100,000 single cells derived from CTR1-, NBS1- and NBS8 organoids were used. Single cell suspension was obtained using a Papain dissociation kit (Worthington) and adhering to the manufacturer’s instructions. Cell pellet was fixed for 20 min in the dark in fixation buffer (Biolegend, San Diego, CA, USA) followed by permeabilization with permeabilization buffer (Biolegend). Pelleted cells by centrifugation for 5 min ad 300× *g* were stained for 20 min in the dark with the fellowing antibodies: mouse anti-SOX2 (1:200, CST #3579S) mouse anti-βIII-tubulin (1:200, CST #TU-20), mouse anti-KI67 (1:200, CST #9449) and guinea pig anti-DCX (1:200, Sigma #AB2253). The cell pellet was washed with permeabilization buffer and incubated with the appropriate secondary antibody Alexa488 and Alexa647 (1:500, Thermo Fisher Scientific). Alexa488 and Alexa647-coupled IgG were used as negative control. Cell pellet was resuspended in PBS/2mM EDTA/0.5%BSA and cell fluorescence was measured using CytoFLEX S (Beckman Coulter). Flow cytometry data were analyzed using FlowJo X v10.6.1 software (FlowJo LLC, Ashland, OR, USA).

### 2.10. Karyotyping and Chromosomal Microarray

The karyotype analysis of NBS-iPSCs was carried out by the Institute of Human Genetics and Anthropology, Heinrich-Heine-University, Düsseldorf, Germany. Genomic DNA from NBS- and NBS8-iPSC lines was extracted using the DNeasy Blood & Tissue Kit (Qiagen, Hilden, Germany) and chromosomal microarray was performed using the Illumina HumanOmni2.5Exome-8 BeadChip v1.3chip at Life&Brain GmbH, Bonn. Genotype and copy number variation (CNV) analysis was performed using Illumina GenomeStudio V2.0.2 (Illumina).

### 2.11. Analysis of Mutational Status of TP53: Library Preparation and Massive Parallel Sequencing

DNA was quantified by a custom-made qPCR assay (Primer for: 5′ AAACGCCAATCCTGAGTGTC-3′; Primer rev: 5′ CATAGCTCCTCCGATTCCAT-3′). Library preparation was carried out using Ion AmpliSeq™ Library Kit 2.0 and Ion AmpliSeq™ Colon and Lung Cancer ResearchPanel v2 with 10 ng of amplifiable DNA following manufacturer’s recommendations. Ion Xpress™ Barcode Adapters Kits were used for barcoding the libraries. Afterwards, libraries were quantified by qPCR using Ion Library TaqMan™ Quantitation Kit on a StepOnePlus™ Real-Time PCR System and were compiled equimolarly for subsequent sequencing reaction. Massive parallel sequencing was conducted on an Ion S5 System using the Ion 520™ & Ion 530™ Kit-OT2 with an Ion 530™ Chip. Primary data analyses were performed by Ion Torrent Suite Software. For variant annotation generated Binary Alignment Map (BAM), files were uploaded to and analyzed by Ion Reporter™ Software using recommended analysis parameter for the Ion AmpliSeq™ Colon and Lung Cancer ResearchPanel v2. Detected variants were examined using the Integrative Genomics Viewer (IGV) [36,37]. All reagents and software were from Thermofisher (Darmstadt, Germany). Selected parts of Exon 2, Exon 4–8 and Exon 10 from the *TP53* gene (NM_000546.5) are covered by the Ion AmpliSeq™ Colon and Lung Cancer ResearchPanel v2 including following amino acids: Ex 2: Met1–Ser20; Ex 4: Glu68–Gly112; Ex 5: Tyr126–Ala138 & Ser149–Gly187; Ex 6: Gly187–Pro223; Ex 7: Val225–Glu258; Ex 8: Asn263–Ala 307; Ex 10: Ile332-Ser367.

### 2.12. Bisulfite Genomic Sequencing

Bisulfite conversion of 500 ng of DNA of CTR1, CTR2, NBS1 and NBS8 organoids at day 20 was conducted using the EpiTec Kit (Qiagenpro) as described [38]. PCR primers for specific amplification of *NNAT* (NG_009263.1) promotor are listed in Table S5. The amplification conditions were denaturation at 95 °C for 13 min, followed by 35 cycles of 95 °C for 60 s, 51 °C for 50 s, and 72 °C for 25 s. PCR reactions were performed using 25 ng bisulfite converted DNA using GoTaq^®^ DNA Polymerase (Promega, Madison, WI, USA). The TA Cloning Kit (Invitrogen) was used for cloning of the amplification product (281 bp) according to the manufacturer’s instructions. Sanger sequencing was performed at the BMFZ (Biomedizinisches Forschungszentrum) core facility of the Heinrich-Heine Universität, Düsseldorf. 12 clones were sequenced to obtain the methylation profile per sample. Analysis of methylated CpGs and methylation graphs were obtained using QUMA (http://quma.cdb.riken.jp/, accessed on 27 June 2020) [39] software.

### 2.13. Statistical Analysis

Statistical analysis was performed with GraphPad Prism Software version 8.02 (GraphPad software, San Diego, CA, USA). An assessment of normality of the data were performed using the Shapiro–Wilk Test. The data follows a normal distribution as *p* > 0.05. Ordinary one-way ANOVA was used for statistical significance analysis for comparisons of the mean among groups, followed by a post hoc test with the use of Dunnett’s multiple comparison test. Statistical significance was assumed at *p* < 0.05, ** *p* < 0.01 and *** *p* < 0.001 and **** *p* < 0.0001. All data are expressed as mean ± 95% confidence interval (qRT-PCR data) or mean ± standard deviation (SD). N and *p*-values are reported in each figure legend.

## 3. Results

### 3.1. iPSCs-Derived from NBS Patients Exhibit Chromosome Instability

To facilitate the generation of an in vitro model system to study NBS, we used previously generated iPSC lines from two NBS patients, referred here as NBS1 and NBS8-iPSCs [19,23]. NBS1-iPSCs carry the homozygous 657del5 mutation [23] and NBS8-iPSCs the heterozygous 657del5 mutation (Supplementary Data 1). Although NBS8-iPSCs carry the heterozygous *NBN* 657del5 mutation, these cells do not express full-length Nibrin [19]. As NBS is characterized by chromosomal instability, genome integrity evaluation was performed at the onset of the current study. While NBS1-iPSCs presents a normal karyotype, copy number variations (CNV) analysis using chromosomal microarrays showed that although no large chromosomal aberrations were not identified, there is a loss of heterozygosity (LOH) on chromosome 8q, where *NBN* is located (Supplementary Figure S1A,B). Of note, the detected LOH is also present in the parental fibroblasts. Karyotype analysis of NBS8-iPSCs revealed a higher number of acquired CNVs and cytogenetic re-arrangements, namely a duplication of most of the long arm of chromosome 5 and a duplication of the telomeric end of chromosome 3p (Supplementary Figure S1C,D). These aberrations were acquired during the reprogramming process, as NBS8-iPSC line was generated by retroviral-based reprogramming. Supplementary Table S1 presents the detailed information about the iPSC lines used in this study. As controls, HFF-derived iPSCs [24], here referred as CTR1-iPSCs and urine cell-derived iPSCs [22], here referred as CTR2-iPSCs, were used.

### 3.2. NBS-iPSCs Efficiently Differentiate into Cerebral Organoids and Recapitulate the Microcephaly Phenotype

**Figure 1:**
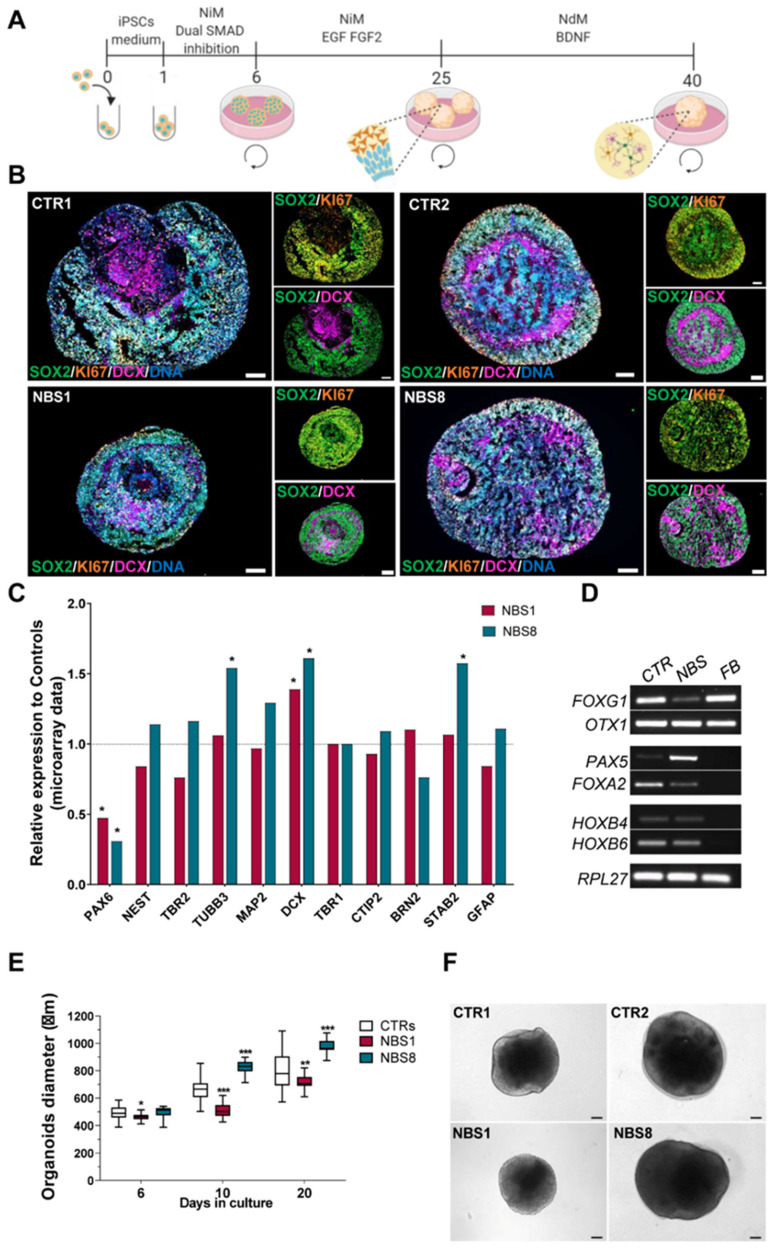
Generation and characterization of NBS cerebral organoids. (**A**) Schematic outline of the main stages of the differentiation protocol to generate the iPSC-derived cerebral organoids. (**B**) Representative immunocytochemistry images of the distribution of cells expressing SOX2, KI67 and DCX in cerebral organoids at day 20. Scale bars, 100 µm. (**C**) Relative mRNA expression analysis of progenitor markers (PAX6, NES and TBR2), pan-neuronal makers (TUBB3, MAP2, DCX), early born neurons (TRB1, CTIP2), late-born neurons (BRN2 and STAB2) and the astrocytes marker GFAP in NBS organoids (NBS1 and NBS8) compared to control organoids (CTR1 and CTR2). (**D**) RT-PCR analysis for brain region specificity at day 20 in control and NBS organoids (forebrain: FOXG and OTX1; midbrain: PAX5 and FOXA2 and hindbrain: HOXB4 and HOXB6). FB, fetal brain control. (**E**) Comparison of the diameter of control, NBS1 and NBS8 cerebral organoids at day 6, 10 and 20. *n* = 76 at day 6, *n* = 61 at day 10 and *n* = 55 at day 20 for both CTR1 and CTR2; *n* = 15 at day 6, *n* = 22 at day 10 and *n* = 18 at day 20 for NBS1 and *n* = 36 at day 6, *n* = 35 at day 10 and *n* = 33 at day 20 for NBS8 cerebral organoids from two independent differentiations. The diameter was significantly smaller in NBS1 organoids. Significance in comparison to control (CTR1 and CTR2) was calculated with one-way ANOVA followed by Dunnett’s multiple comparison test; * *p* < 0.05, ** *p* < 0.01, *** *p* < 0.001. (**F**) Representative bright-field images of control and NBS cerebral organoids at day 20. NBS1 organoids visually lack neuroepithelial structures. Scale bars, 100 µm.

To address how NBS affects early human neurodevelopment and to dissect the mechanisms underlying microcephaly in NBS patients, we implemented a published suspension protocol to generate forebrain organoids [25] (Figure 1A). These organoids are composed of neural progenitor cells (NPCs) self-organized into ventricular zone (VZ) -like structures surrounded by outer layers of early born and mature neurons. By day 20, immunohistochemical analysis revealed that control and NBS organoids have an internal cyto-architecture composed mostly of the SRY-Box Transcription Factor 2 (SOX2)^+^ NPCs aligned in the (VZ)-like structure and surrounded by doublecortin (DCX)^+^ early born neurons (Figure 1B). To evaluate the efficiency of the differentiation, we assessed mRNA levels of progenitor markers, pan-neuronal makers, early born neurons, late-born neurons and the astrocyte marker *GFAP*. While there is heterogeneity in the expression of the neuronal markers between NBS1 and NBS8 organoids, neuronal progenitor markers such as *PAX6* were significantly down-regulated in all NBS organoids. On the other hand, compared to controls, NBS organoids showed an up-regulation of the neuronal markers *DCX* and *TUBB3* (Figure 1C). To identify the regional specificity of the cerebral organoids, we performed a RT-qPCR for forebrain, midbrain and hindbrain markers. Despite the use of a protocol that was developed to yield region-specific forebrain organoids, both 20 days control and NBS cerebral organoids expressed not only the forebrain markers *FOXG1* and *OTX1*, but also the midbrain markers *PAX5* and *FOXA2* and the hindbrain markers *HOXB4* and *HOXB6*, albeit at lower expression levels (Figure 1D). To analyze the developmental stage of the cerebral organoids with respect to human fetal brain, we compared the transcriptome profiles of CTR1, CTR2, NBS1, and NBS8 organoids at day 20 to the transcriptomic data from the Allen Human Brain Atlas (https://www.brainspan.org, accessed on 8 May 2020) [40]. Our day 20 cerebral organoids are at a developmental stage close to 8–9 post-conception week (pcw) fetal brains (Supplementary Figure S2A). To test whether NBS organoids recapitulate the microcephaly phenotype, we evaluated the size of the organoids over a period of 20 days. Although the size of NBS8 organoids was similar to that of controls at day 6, the organoids became significantly larger after prolonged culture. On the other hand, NBS1 organoids were significantly smaller in comparison to controls and the size was not compensated during the time in culture. (Figure 1E). In addition to the smaller size, NBS1 organoids were relatively spherical and visually lacked the neuroepithelial structures observed in the controls and NBS8 organoids (Figure 1F). Together these results imply that control and NBS-iPSCs efficiently differentiate into cerebral organoids, which recapitulate aspects of early human brain development with defined molecular markers. Furthermore, NBS1 organoids, carrying the homozygous *NBN* mutation, recapitulate the microcephaly phenotype.

### 3.3. NBS Organoids Present a Disrupted Cytoarchitecture with Normal Proliferation of the NPCs

**Figure 2:**
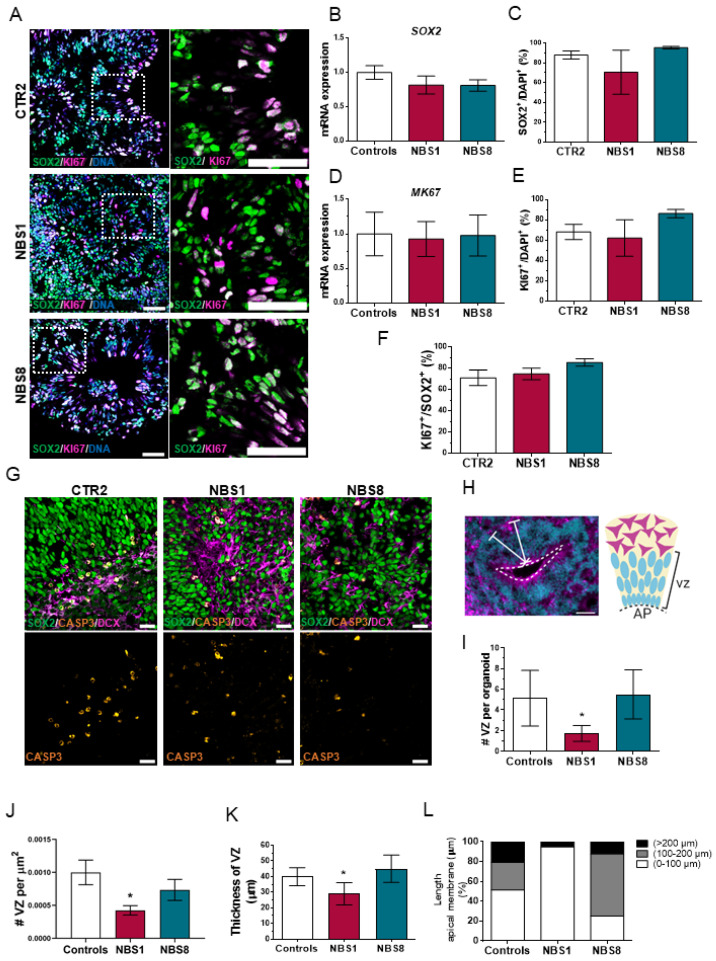
Analysis of proliferation of the NPCs and VZs cytoarchitecture at day 20. (**A**) Representative confocal pictures of immunostainings for SOX2 and KI67 in CTR2, NBS1 and NBS8 organoids. Scale bars, 50 µm. (**B**) qRT—PCR analysis of *SOX2* mRNA expression in NBS1 and NBS8 organoids relative to control organoids. (**C**) Quantification of the SOX2-positive cells in CTR2 and NBS1 and NBS8 organoids. (**D**) qRT—PCR analysis of *KI57* mRNA expression in NBS1 and NBS8 organoids relative to control organoids (CTR1 and CTR2). (**E**) Quantification of the KI67-positive cells in CTR2 and NBS1 and NBS8 organoids. (**F**) Quantification of the KI67-positive cells within the SOX2-positive cells in CTR2, NBS1 and NBS8 organoids. (**G**) Representative confocal pictures of immunostainings for SOX2, cleaved-CASP3 and DCX in CTR2, NBS1 and NBS8 organoids. Cleaved-CASP3 colocalized with the DCX positive cells. Scale bars, 50 µm. (**H**) Schematic illustration of a ventricular zone (VZ). (**I**,**J**) Quantification of the number of the VZs (**I**) per organoid and (**J**) per area (µm^2^) in control (CTR1 and CTR2) and NBS1 and NBS8 organoids. (**K**) Quantification of the thickness of the VZs in µm in control (CTR1 and CTR2) and NBS1 and NBS8 organoids. (**L**) Quantification of the length of the apical membrane per VZ in control, NBS1 and NBS8 organoids; 3 organoids from three independent differentiations were analyzed. (**B**,**D**) Results are mean ± 95% confidence interval derived from three independent differentiations. (**C**–**F**,**I**,**J**) Results are mean ± SD derived from three organoids from three independent differentiations. Significance in comparison to control was calculated with one-way ANOVA followed by Dunnett´s multiple comparison test. * *p* < 0.05.

NPCs possess incredible plastic features and an enhanced capacity to sense the environment. Furthermore, they can divide both symmetrically and asymmetrically, stay quiescent for long periods, and undergo active proliferation or differentiate upon instruction. Environmental cues can trigger the proliferation of NPCs or the acquisition of a terminally differentiated phenotype [41]. We analyzed the expression of the NPCs marker SOX2 together with the proliferation marker KI67 in our cerebral organoids (Figure 2A). At day 20 both control and NBS organoids were mainly composed of SOX2^+^ NPCs, with no significant differences in the total number of SOX2^+^ cells (Figure 2C) as well as in the *SOX2* mRNA expression (Figure 2B) between control and NBS organoids. Furthermore, a similar proliferation of the NPCs was observed based on KI67 expression (Figure 2D–F). Next, we examined if the reduction in size observed in NBS1 organoids was due to enhanced cell apoptosis in the cerebral organoids. We found a comparable number of cleaved Caspase-3^+^ cells between control and NBS organoids, mostly located outside the VZ and co-localized with the DCX^+^ cells (Figure 2G). Lastly, we explored the internal cyto-architecture of the cerebral organoids by determining the number and the thickness of the VZ, as well as the length of the apical membrane. Control organoids displayed typical VZ containing SOX2^+^ NPCs proliferating at the apical surface of the ventricular zone (Figure 2H), while NBS1 organoids exhibited a markedly reduction of these well-defined VZs (Figure 2I,J). Likewise, the VZs were significant smaller (Figure 2K) with smaller apical membranes (Figure 2L). Notably, at day 20 there were no clear differences in the VZs from the NBS8 organoids compared to controls. Our results suggest that although the population of the NPCs is not affected in an early developmental stage, NBS1 organoids carrying the homozygous *NBN* mutation exhibited disrupted cyto-architecture that can affect normal brain development.

### 3.4. NBS1 and NBS8 Organoids Show a Distinct Transcriptomic Profile

**Figure 3:**
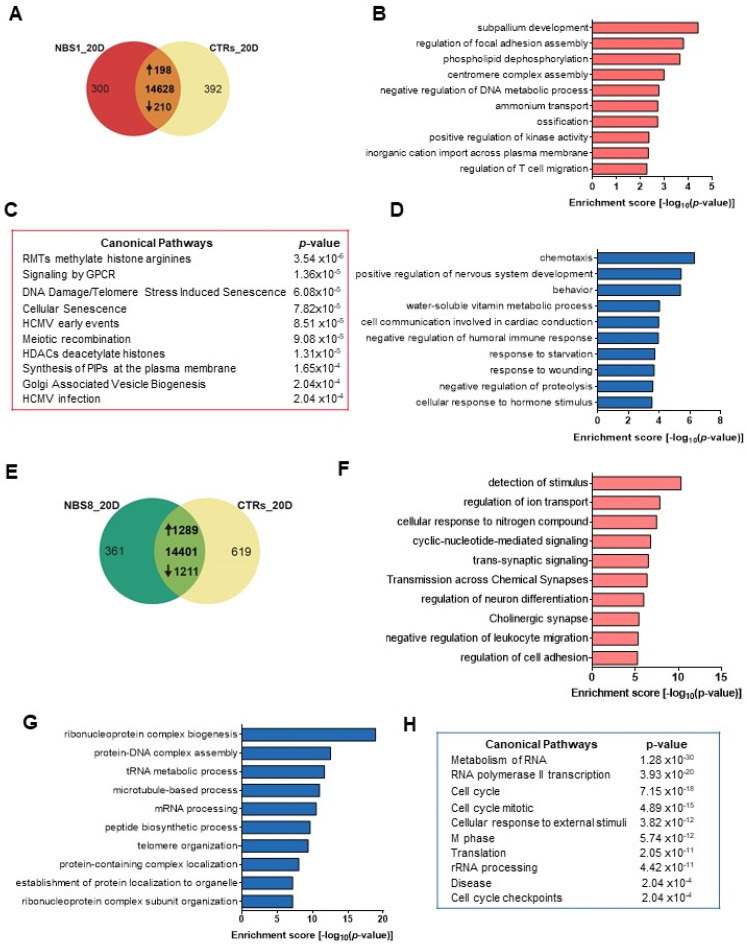
Global transcriptome functional analysis of control and NBS organoids at day 20. (**A**) Venn diagram showing genes expressed only in NBS1 organoids (300), in control organoids (392) and common to both (12,828) (detection *p* value < 0.05). (**B**,**C**) Enrichment clustered GOs (**B**) and canonical pathways enrichment analysis (**D**) of the of the significantly up-regulated 198 genes in NBS1 organoids compared to control organoids (Top 10 ranked). (**D**) Canonical pathways enrichment analysis of the of the significantly down-regulated 210 genes in NBS1 organoids compared to control organoids (Top 10 ranked). (**E**) Venn diagram showing genes expressed only in NBS8 organoids (361), in control organoids (619) and common to both organoids (14,401; detection *p* value < 0.05). (**F**) Bar chart of the enriched clustered GOs (Top 10 ranked) of the significantly up-regulated 1289 genes in NBS8 organoids compared to control organoids. (**G**,**H**) Enrichment clustered GOs (**G**) and canonical pathways enrichment analysis (**H**) of the of the significantly down-regulated 1211 genes in NBS8 organoids compared to control organoids (Top 10 ranked).

To gain further insights into the pathophysiology of NBS, we performed transcriptome analysis of both control and NBS organoids at day 20 of differentiation and identified differentially expressed genes (DEGs). Hierarchical clustering revealed one cluster containing the NBS8 organoids and the second cluster containing CTR1, CTR2 and NBS1 organoids, thus indicating that NBS8 organoids have a distinct transcriptome profile (Supplementary Figure S2B,C). To further interrogate the DEGs, we compared the transcriptome of the NBS1 and NBS8 organoids to both control organoids. We found 198 up-regulated and 210 down-regulated DEGs in NBS1 organoids compared to both control organoids (Figure 3A, Supplementary Table S2). Enrichment analysis of the up-regulated DEGs showed *subpallium development, regulation of focal adhesion assembly, phospholipid dephosphorylation* and *centromere complex assembly* as the most enriched GOs (Figure 3B). Regarding the canonical pathways, up-regulated DEGs were significantly enriched in *RTMs methylate histone arginines, signaling by GPCR, DNA damage/Telomere stress induce senescence* and *cellular senescence* (Figure 3C). Down-regulated genes were enriched for GOs such as chemotaxis, *negative regulation of proteolysis* and *cellular response to hormonal stimuli* (Figure 3D). The same analysis was performed using the NBS8 organoids. One-thousand two-hundred eighty-nine genes were identified as up-regulated compared to both control organoids. (Figure 3E, Supplementary Table S2). Among the enriched GOs, we identified clusters associated with *synaptic signaling* and *regulation of neuron differentiation*, as well as *regulation of ion transport and cell adhesion* (Figure 3F). On the other hand, cluster analysis of the 1211 down-regulated genes identified *ribonucleoprotein complex biogenesis, protein-DNA complex assembly, microtubule-based process and telomere organization* as the most significantly enriched GOs (Figure 3G). The analysis of the canonical pathways identified the expression levels of genes involved in *Metabolism of RNA, RNA polymerase II transcription, Cell cycle, Cell cycle mitotic* and *Cell cycle checkpoints* pathways to be significantly reduced (Figure 3H). Inherently, NBS1- and NBS8 organoids show variable modulation of genes associated with regulation of gene expression such *chromatin assembly*, *DNA replication-dependent nucleosome assembly, negative regulation of gene expression/epigenetic, DNA packing* which were found up-regulated in NBS1 and down-regulated in NBS8. Interestingly, both NBS1 and NBS8 organoids showed down-regulated genes associated with behavior and cognition (Supplementary Table S2). Our results indicate heterozygous and homozygous carriers of the *NBN* 657del5 mutation result in slightly distinct phenotypes.

### 3.5. NBS Organoids Show Accumulation of DNA Damage

**Figure 4:**
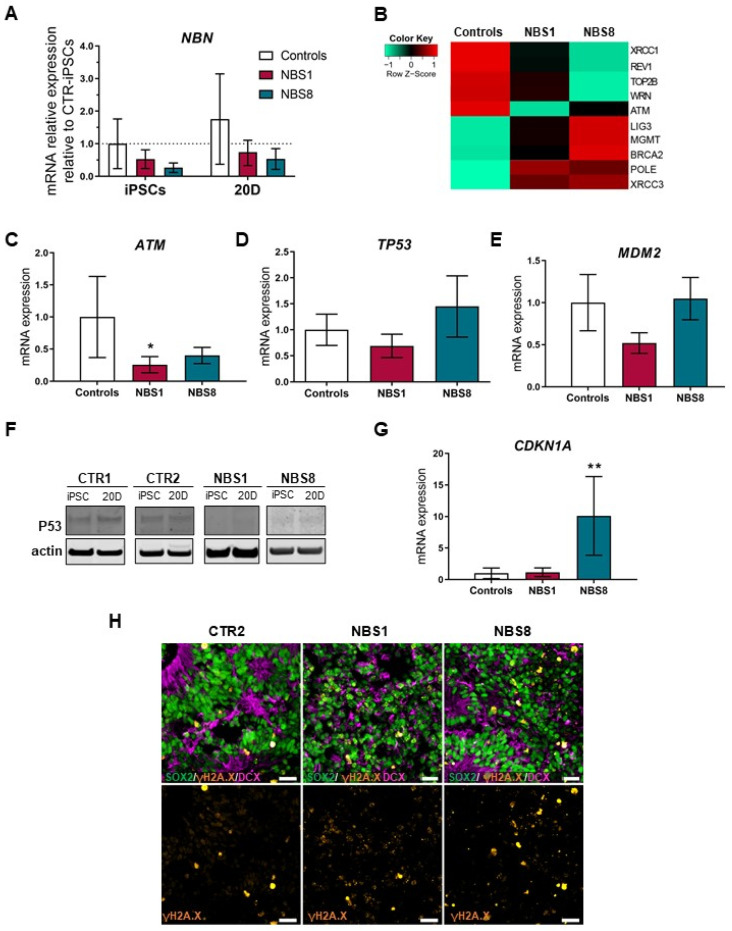
DNA damage response analysis in NBS organoids. (**A**) qRT-PCR analysis of the C-Terminal fragment of *NBN* in NBS1- and NBS8- iPSCs and 20D organoids compared to control iPSCs (CTR1 and CTR2) and 20D control (CTR1 and CTR2) organoids. (**B**) Heatmap showing differential gene expression analysis of selected DNA damage repair-related genes expressed in control and NBS organoids at day 20. (**C**,**D**) qRT-PCR analysis of *ATM* (**C**) and *TP53* (**D**) mRNA expression in NBS1 and NBS8 organoids relative to control organoids (CTR1 and CTR2). (**F**) Immunoblotting for total p53 in CTR1, CTR2 and NBS1 and NBS8 iPSCs and 20 days cerebral organoids. (**E**,**G**) qRT-PCR analysis of *MDM2* (**E**) and *CDKN1A* (**G**) mRNA expression in NBS1 and NBS8 organoids relative to control organoids (CTR1 and CTR2). (**H**) Representative confocal pictures of immunostainings for SOX2, phosphorylated histone H2A.X and DCX in CTR2-, NBS1- and NBS8 organoids. NBS organoids display an increase in γH2A.X nuclear foci formation. Scale bars, 50 µm. (**A**,**C**–**E**,**G**) Results are mean ± 95% confidence interval derived from three independent differentiations. Significance in comparison to control was calculated with one-way ANOVA followed by Dunnett´s multiple comparison test. * *p* < 0.05, ** *p* < 0.01.

Defective DNA damage repair is a well-established cellular feature observed in NBS patients [42]. However, the precise mechanism as to how NBS cells respond to DSBs and how it affects brain development is poorly understood. Using primers located in the C-terminal of *NBN*, we observed that the C-terminal transcript in NBS-iPSCs and NBS organoids is down-regulated but not completely abolished (Figure 4A). In view of this, we took a look at the DNA damage response at day 20 of our cerebral organoids. At this time-point, we found de-regulation in the expression of genes involved in the DDR pathway (Figure 4B). Down-regulation of *ATM* was observed, which was confirmed by qRT-PCR (Figure 4C). Interestingly, expression of *TP53* was not significantly regulated (Figure 4D). However, when the protein level was analyzed, NBS organoids clearly showed a reduction in p53 levels compared to control organoids (Figure 4F). To examine whether the decrease in p53 expression was due to the presence of mutations, *TP53* target sequencing was carried out in order to identify potential disease-causing variants. No mutations were found in control or NBS organoids (data not shown). As MDM2 is a key regulator of p53, *MDM2* mRNA expression was analyzed and no significant differences between control and NBS organoids were found (Figure 4E). Surprisingly, NBS8 organoids expressed significantly higher levels of *CDKN1A*, the major p53 target which controls cell cycle arrest after DNA damage [43]. Outstandingly, high levels of DSBs were observed in the NBS organoids, judged by the increase of γ-H2AX nuclear foci formation co-localized with the SOX2^+^ NPCs (Figure 4H). Our results indicate that NBS organoids display an impaired DDR pathway, with the accumulation of DNA damage and subsequent genome instability probably due to the lower levels of p53 suggesting that p53 plays a central role in orchestrating the fate of NPCs in NBS organoids.

### 3.6. NBS Organoids Exhibit Premature Differentiation Accompanied by NNAT Over-Expression

**Figure 5:**
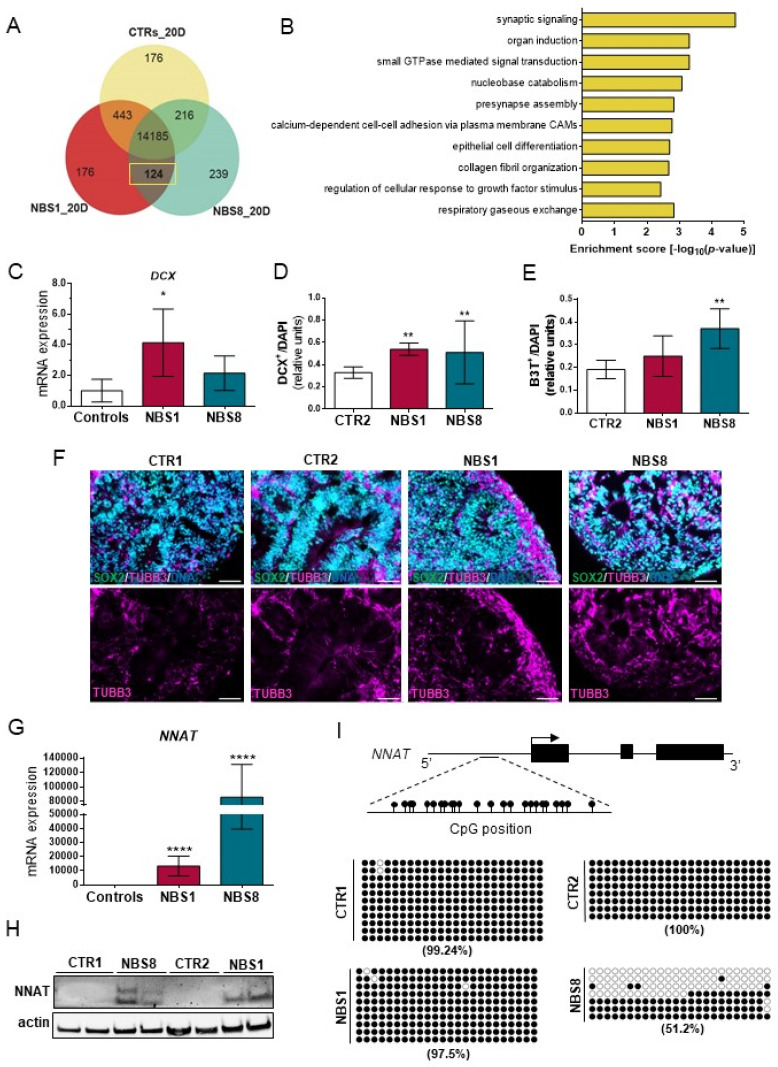
Neurodifferentiation propensity of NBS organoids. (**A**) Venn diagram showing the exclusively expressed genes in NBS1 and NBS8 organoids (124) compared with in control organoids (detection *p* value < 0.05). (**B**) Bar chart of the enriched clustered GOs (Top 10 ranked) of the exclusively expressed genes (124) NBS1 and NBS8 organoids compared to control organoids. (**C**) qRT-PCR analysis of *DCX* mRNA expression in NBS1 and NBS8 organoids relative to control organoids (CTR1 and CTR2). (**D**,**E**) Quantification of the DCX- (**D**) and βIII-Tubulin^+^ (**E**) positive cells in 20 days CTR2, NBS1 and NBS8 organoids. Results are mean ± SD from three organoids from three independent differentiations. ** *p* < 0.01. (**F**) Representative pictures of immunostainings of SOX2 and βIII-Tubulin^+^ in CTR1 CTR2, NBS1, and NBS8 organoids showing an increase in βIII-Tubulin^+^ cells. (**G**) qRT-PCR analysis of *NNAT* mRNA expression in NBS1 and NBS8 organoids relative to control organoids (CTR1 and CTR2). (**H**) Immunoblotting for NNAT in CTR1 and NBS8 organoids at day 20 and CTR2 and NBS1 organoids at day 40. (**C**,**G**) Results are mean ± 95% confidence interval from three independent differentiations. Significance in comparison to control was calculated with one-way ANOVA followed by Dunnett´s multiple comparison test. * *p* < 0.05, **** *p* < 0.0001. (**I**) Bisulfite sequencing of a CpG island within the promotor region of *NNAT*. The % methylated CpG dinucleotides in CTR1, CTR2, NBS1 and NBS8 organoids at day 20 is shown. Filled circles denote methylated CpG dinucleotides. White circles denote unmethylated CpGs.

To gain deeper insights into the molecular portraits of NBS-regulated gene expression, we analyzed the exclusively expressed genes between NBS1 and NBS8 organoids compared to the control. We identified 124 genes expressed exclusively in the NBS- and not in the control organoids (Figure 5A). Functional enrichment analysis revealed that most of these genes are involved in *synaptic signaling*, *pre-synapse assembly* and *organ induction* (Figure 5B). In addition to the genes directly linked with neuronal development, we identified genes involved in the *regulation of interleukin-6 production* and matrisome-associated, including regulators of the ECM and secreted factors. As previously described, NBS organoids showed up-regulation of several neuronal differentiation markers (Figure 1C). To evaluate the differentiation propensity of NPCs, we analyzed the expression of the neuronal marker DCX. Although mRNA expression was only up-regulated in the NBS1 organoids (Figure 5C), both NBS1 and NBS8 organoids showed a significantly higher number of DCX^+^ cells (Figure 5D), thus suggesting premature neuronal differentiation. Likewise, NBS organoids showed an increase in the number of βIII-Tubulin^+^ cells, but more pronounced in the NBS8 organoids (Figure 5E,F). Interestingly, we identified *neuronatin* (*NNAT*)—a paternally imprinted gene involved in brain development as the most up-regulating genes in day 20 NBS organoids. *NNAT* mRNA expression was barely detectable in the control organoids and highly up-regulated in NBS organoids (Figure 5G). Consistent with the mRNA expression, NNAT protein was only present in NBS organoids (Figure 5H). Regulation of *NNAT* expression depends on the degree of *NNAT* methylation [44]. Thus, we analyzed the methylation status within a CpG island within the promoter region. We found high levels of methylation in this region in the control organoids (CTR1 = 99.24% and CTR2 = 100%) and NBS1 organoids (97.5%). However, a markedly decrease in the methylation at this CpG island was observed in NBS8 organoids (51.2%). Collectively, our results suggest that NBS organoids undergo premature neurogenesis governed in part by NNAT and this abnormal over-expression is controlled by the loss of methylation in a regulatory region within the *NNAT* promoter.

### 3.7. NBS Organoids at Day 40 Acquire an Abnormal Regulation of Cell Cycle

**Figure 6:**
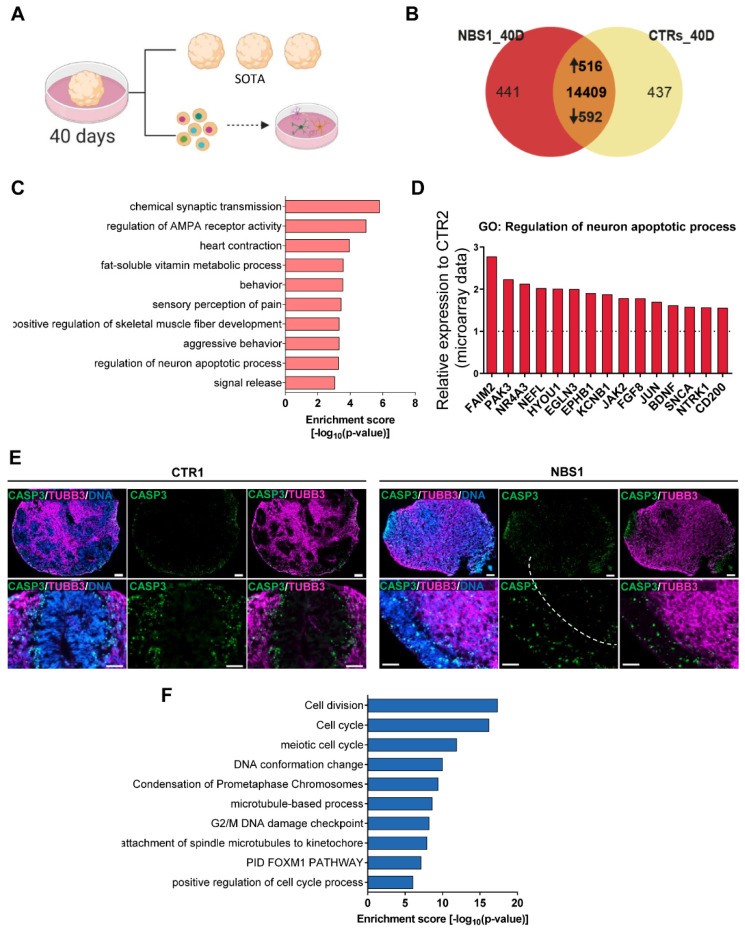
NBS organoids profile at day 40. (**A**) Schematic depicting the analysis of the NBS and control organoids at day 40. SOT-analysis was performed. Additionally, cerebral organoids were dissociated into single cells for FACS analysis and re-plating in 2D. SOT: single organoid transcriptome. (**B**) Venn diagram showing genes expressed only in NBS1 organoids (441), in control organoids (437) and common to both at day 40 (14,409; detection *p* value < 0.05). (**C**) Bar chart of the enriched clustered GOs and Pathways (Top 10 ranked) of the up-regulated genes (1516) in NBS1- organoids compared to control organoids at day 40. (**D**) mRNA expression of genes part of the GO: Regulation of neuron apoptotic process in NBS1- compared to CTR2 organoids at day 40. Gene expression extracted from the SOT-analysis. (**E**) Representative pictures of immunostainings of cleaved-CASP3 and βIII-Tubulin in CTR1 and NBS1 organoids showing increased apoptosis in NBS1 organoids. (**F**) Bar chart of the enriched clustered GOs (Top 10 ranked) of the down-regulated genes (592) in NBS1- organoids compared to control organoids at day 40.

To further investigate the consequences of NBS in postmitotic neurons, we differentiated the cerebral organoids for 40 days and performed single organoid transcriptome analysis (SOT-analysis). To ensure the maturity of the organoids, dissociation into single cells for further FACS analysis and re-plating in 2D was performed (Figure 6A). As before, we examined the expression of SOX2, βIII-Tubulin, DCX and KI67. Compared with control organoids, NBS organoids were composed of fewer SOX2^+^ cells (NBS = 45.97%; control = 59.48%). On the other hand, the population of βIII-Tubulin^+^ cells was slightly higher (NBS = 35.15%; control = 28.6%), suggesting a more differentiated population in NBS organoids. Around 95% of the NBS organoids are composed of DCX^+^ cells, were 20% of these cells are proliferating as indicated by the presence of DCX^+^/KI67^+^ cells (Supplementary Figure S3A–C). After re-plating in 2D, we could still observe the presence of SOX2^+^ NPCs and a neuronal network formed by the βIII-Tubulin^+^ neurons (Supplementary Figure S3D), further demonstrating the integrity of our cerebral organoids after 40 days of differentiation. SOT-analysis identified 516 up-regulated and 592 down-regulated genes in NBS1 organoids compared to control organoids (CTR1 and CTR2) (Figure 6B, Supplementary Table S3). Analysis of the enriched GOs showed a persistent up-regulation of chemical synaptic transmission and regulation of AMPA receptor activity in the NBS1 organoids (Figure 6C). Interestingly, up-regulation of *FAIM2, PAK3, NR4A3, NEFL, HYOU1, EGLN3, EPHB1, KCNB1, JAK2, FGF8, JUN, BDNF, SNCA, NTRK1*, and *CD200* involved in the regulation of neuron apoptotic processes was observed (Figure 6D). We therefore evaluated the presence of apoptosis by immunostaining for cleaved CASP-3 together with the neuronal marker βIII-Tubulin in the cerebral organoids. At day 40, we detected a few apoptotic cells in control organoids in the areas where βIII-Tubulin-positive neurons reside. However, NBS organoids moreover the unorganized cyto-architecture observed with the lack of the VZ, the number of cleaved CASP-3^+^ cells was high. Interestingly, these apoptotic cells were distributed throughout the entire organoid, affecting not only the βIII-Tubulin^+^-neurons but also the NPCs (Figure 6E). Interestingly, among the down-regulated DEGs, transcripts related to *cell division*, *cell cycle*, *DNA G2/M DNA damage checkpoint* were identified (Figure 6F). The transcripts with reduced expression include *CHEK1*, *NBN*, *MRE11*, *CCNA2*, *CDK1*, *CDC42*, *CCNB1*, *CDKN2C* and *RAD9B*. Furthermore, *DNA repair*, *regulation of TP53 activity* were also observed as down-regulated GOs (Supplementary Table S3). Taken together, our results imply that NBS organoids undergo premature neurodifferentiation along with increased apoptosis. Furthermore, SOT-analysis points to a de-regulation of cell cycle as the pivotal mechanism underlying perturbed neurodevelopment in the NBS organoids.

### 3.8. Bleomycin-Induced Cytotoxicity Highlights the Aberrant NBS Phenotype

**Figure 7:**
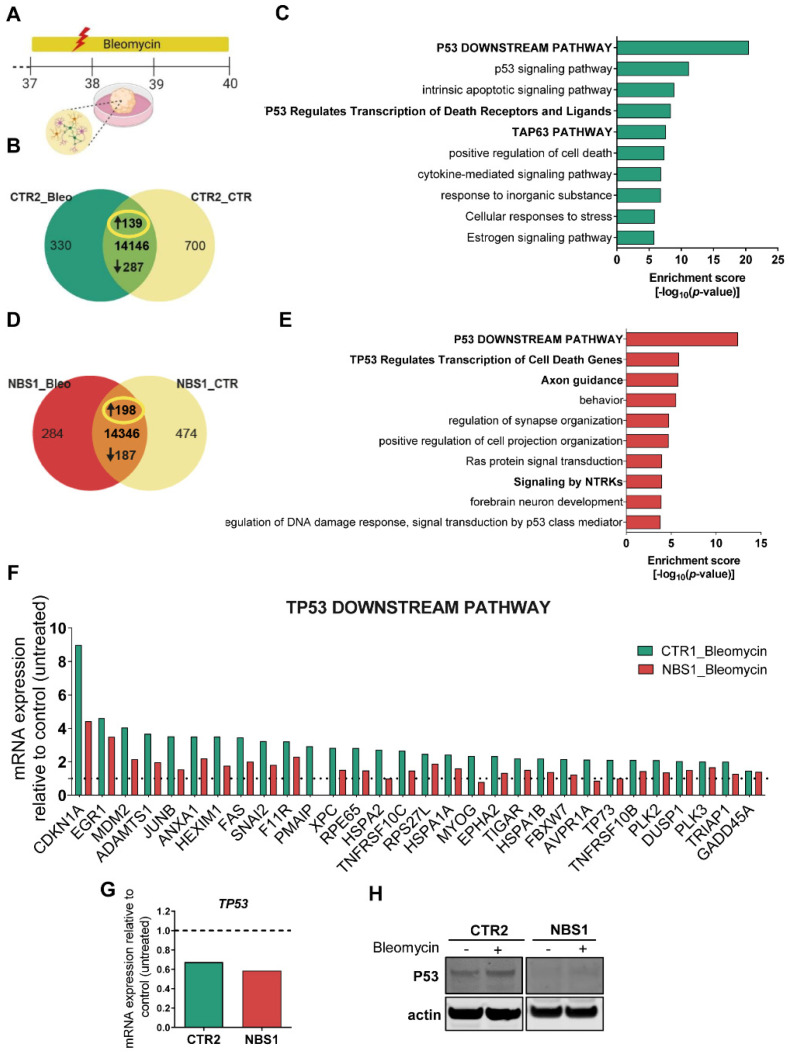
Effects of bleomycin in NBS organoids at day 40. (**A**) Schematic depicting the strategy to induce DNA damage with Bleomycin treatment during 72 h from day 37 to day 40 of CTR2- and NBS1 organoids. (**B**) Venn diagram showing genes expressed only in CTR2_Bleomycin organoids (330), in CTR2_control organoids (700) and common to both (144,146; detection *p* value < 0.05). (**C**) Bar chart of the enriched clustered GOs and Pathways (Top 10 ranked) of the up-regulated genes (139) in CTR2_Bleomycin organoids compared to CTR2_control organoids (**D**) Venn diagram showing genes expressed only in NBS1_Bleomycin organoids (284), in NBS1_control organoids (474) and common to both (414,346; detection *p* value < 0.05). (**E**) Bar chart of the enriched clustered GOs and Pathways (Top 10 ranked) of the up-regulated genes (198) in NBS1_Bleomycin organoids compared to NBS1_control organoids. (**F**) mRNA expression of the genes part of the up-regulated TP53 downstream pathway in CTR2_Bleomycin organoids and NBS1_Bleomycin organoids compared to CTR2_control organoids and NBS1_control organoids, respectively. All genes were significantly up-regulated in CTR2 after bleomycin treatment. (**G**) mRNA expression of *TP53* in CTR2_Bleomycin organoids and NBS1_Bleomycin organoids compared to CTR2_control organoids and NBS1_control organoids. (**H**) Western blot analyses of total P53 in CTR2 and NBS1 organoids after bleomycin treatment.

To understand the effects of how increased accumulation of DNA damage affects brain development in NBS patients, we subjected the cerebral organoids to bleomycin treatment over a period of 72 h with subsequent SOT-analysis. (Figure 7A). Evaluation of DEGs in control organoids after bleomycin treatment revealed an up-regulation of 139 genes (Figure 7B, Supplementary Table S4). The subsequent functional enrichment analysis showed that these genes are part of the *P53 downstream pathway* and *TP53 regulates transcription of cell death receptors and ligands (*Figure 7C, Supplementary Table S4), thus highlighting bleomycin-induced DNA damage as with a significant effect on the p53 signaling pathway in control organoids. Focusing on NBS organoids, 198 up-regulated genes were identified when comparing bleomycin treatment to control conditions (Figure 7D, Supplementary Table S4). Interestingly, these genes were also enriched for P53 downstream pathway. TP53 regulates transcription of cell death genes. Furthermore, bleomycin treatment reinforces axon guidance, regulation of synaptic organization, and forebrain development, already exacerbated in basal conditions in the NBS organoids (Figure 7E). We next evaluated the levels of mRNA expression of the genes up-regulated within the TP53 downstream pathway between control and NBS organoids after bleomycin treatment. While both CTR2 and NBS1 organoids activate the p53 signaling pathway to mediate DDR, NBS organoids showed an impairment in the activation of these genes compared to CTR2 organoids, as observed by the significantly lower expression of these genes which include *CDKN1A*, *FAS* and *GADD45A* (Figure 7F). Additionally, the analysis of the DEGs upon bleomycin treatment showed 287 down-regulated genes in control organoids (Supplementary Figure S4A). These genes were highly significantly enriched for cell cycle and cell cycle checkpoints (Supplementary Figure S4B). NBS1 organoids followed the same pattern, with 187 down-regulated genes (Supplementary Figure S4C) being enriched for cell cycle, cell division, Mitotic G1 phase and G1/M transition and DNA Double-strand break repair (Supplementary Figure S4D). Next, we evaluated the expression of the common set of genes between CTR2 and NBS1 organoids which belong to the cell cycle pathway. While CTR2 organoids significantly down-regulated cell cycle-related genes, NBS1 organoids were not able to induce such dramatic changes in gene expression after bleomycin treatment (Supplementary Figure S4E). The consequences of these impairments are correlated with the expression of *MKI67*, where CTR2 organoids drastically reduced *MKI67* levels in contrast to a slight reduction observed in NBS1 organoids (Supplementary Figure S4F). To have a better understanding of the specific effects of bleomycin treatment in NBS organoids, we performed enrichment analysis of the 94 exclusively expressed genes (Supplementary Figure S4G). Bleomycin treatment specifically induced the expression of genes associated with trans-synaptic signaling, glycosphingolipid biosynthesis, leukocyte apoptotic process and neurotransmitter transport in the NBS organoids (Supplementary Figure S4H). Taken together, our results have shown that after exposure to a genotoxic agent, NBS organoids undergo an impairment in P53-regulated pathways and consequently cell cycle regulation due to low levels of p53.

## 4. Discussion

While microcephaly is the hallmark of NBS, the mechanisms that lead to reduced brain size in these patients are largely unknown, mostly due to the hurdles and limitations associated with studying NBS. By generating for the first-time patient iPSC-derived cerebral organoids we were able to investigate cellular and molecular effects of the *NBN* mutation during early neurogenesis.

In this work, two distinct NBS patient-derived iPSC lines carrying the *NBN* 657del5 mutation were analyzed: NBS1 (homozygous) and NBS8 (heterozygous). NBS8-iPSCs present a much higher chromosomal instability, with aberrations acquired during the reprogramming process, as such duplications of chromosome 5q, which can confer growth advantages during the reprogramming process [45]. These differences in the genotype of both iPSC lines can result in distinct phenotypes, as was also observed in several NBS patients [6]. As NPCs and post-mitotic neurons respond differently to endogenous DNA damage [1], we analyzed the cerebral organoids at two time-points: 20 days and 40 days differentiation. Day 20 cerebral organoids correspond to 8–9 pcw of human brain development and are composed mainly of cells with a forebrain identity. However, a sub-population of cells with midbrain and hindbrain identity were also present as observed by others [46].

Microcephaly associated abnormalities are a hallmark of NBS which frequently occur during the early neurodevelopmental stages, although in some patients only develop postnatally [6,47]. While both NBS1 and NBS8 organoids showed a similar expression profile for the analyzed differentiation markers, morphology-based analysis revealed distinct phenotypes. Although an increase in size at day 20 was observed, NBS8 organoids (heterozygous) presented NPCs aligned in the apical membrane of the VZs and neurons in the cortical region similar to control organoids. On the other hand, NBS1 organoids (homozygous) were significantly smaller in size and presented a disrupted architecture with a disorganized distribution of cells, resulting in fewer and smaller VZs, thus recapitulating the microcephaly phenotype. So far, the use of cerebral organoids to model microcephaly has only been performed with iPSC-derived from patients carrying centrosome-related mutations. Apart from the smaller size of these organoids, a depletion of NPCs was observed [17,46,48,49]. Interestingly, neither the number nor the proliferation of NPCs was affected in NBS organoids in comparison to the healthy controls. These results hint at a different mechanism underlying microcephaly in NBS patients. In accordance with our results, Esk et al. recently identified abnormal deregulated VZs integrity and abnormal localization of the NPCs as the mechanism underlying *IER3IP1*-related microcephaly [50].

Transcriptome-based analysis after 20 days of differentiation reinforced that NBS1- and NBS8 organoids respond in distinct manner to the endogenous levels of oxidative stress. NBS1 organoids showed up-regulation of genes involved in epigenetic regulation of gene expression, centromere complex assembly and senescence, implying these cells attempt to maintain genomic and epigenomic integrity since DNA methylation and chromatin remodeling are linked to DNA damage and repair [51]. However, several genes that regulate transcription were found down-regulated in the NBS8 organoids, together with genes associated with cell cycle and cell cycle checkpoints. Following DSBs, ATM is activated by auto-phosphorylation and undergoes spatial relocation to the DSB site, followed by the phosphorylation of H2AX. In turn, H2AX recruits the MRN complex to the DSB, a process which requires NBN. The DNA damage response is then reinforced by further deposition of ATM at the DSB site, promoted by the MRN complex. In addition to sensing DSBs, NBN phosphorylates ATM to control cell cycle checkpoints [52,53]. Among the numerous genes within the DDR pathway de-regulated in the NBS organoids, *ATM* was found significantly down-regulated, in accord with previous studies demonstrating that NBS cells dramatically reduce ATM activation [54].

These normal ATM-dependent signaling events led to a rapid p53 stabilization and transcriptional induction of *CDKN1A*, which encodes p21- a cyclin-dependent kinase inhibitor which triggers G1 cell cycle arrest or a permanent state of senescence or apoptosis [52,53]. NBS organoids showed a dramatic reduction in p53 protein levels, with no differences in *TP53* mRNA expression, consistent with our previous results using NPCs derived from NBS-iPSCs [21]. Strikingly, *Nbn* knockout mice or inducible *Nbn* null mice cells showed an activation of p53 stabilization [16,55,56]. This emphasizes that the use of neuronal cells derived from iPSC-derived from NBS patients can unveil new mechanistic insights underlying NBS pathogenesis. Here we propose that in an early neurodifferentiation phase, p53 is still able to activate the transcription of *CDKN1A* (p21) in the NBS organoids, thus demonstrating that p53 function is not completely abrogated. However, the DDR pathway is compromised, as shown by an increase in γ-H2AX nuclear foci formation in the NPCs of the NBS organoids, thus higher levels of DNA damage. In an attempt to increase the stabilization of p53, we treated the cerebral organoids with nutlin-3a, which functions to inhibit the interaction between MDM2 and p53 [57]. NBS organoids underwent significant up-regulation of *CDKN1A*, leading to a down-regulation of *KI67* and subsequent cell death. Our results indicate that inhibition of MDM2-p53 interaction by nutlin-3a allows p53 stabilization and the enhanced response to nultin-3a further demonstrates that the levels of DNA damage are higher in NBS organoids. An impaired DDR pathway was observed in the NBS organoids, probably due to the attenuation of the ATM-p53 pathway activation after endogenous DNA damage, in line with previous studies [58,59].

Our data suggests this impairment has an impact on the fate of the NPCs, leading to premature differentiation. NBS organoids exclusively expressed genes associated with synaptic signaling and neuronal differentiation as indicated by the increased number of DCX and βIII-tubulin^+^ cells. Our results are in contrast with the previous findings in NBS using NPCs derived from NBS-iPSCs showing delayed neurogenesis [21]. This discrepancy could be due to the different approaches used, such as different differentiation protocols and the iPSC were differentiated only to the NPC stage in Halevy et al. (2016). Premature differentiation of the NPCs leading to its depletion is the common mechanism underlying microcephaly described in patient-derived organoids [17,46,48,49,50]. The accumulation of DNA damage can promote accelerated neurogenesis by increasing the expression of genes that regulate cellular and cognitive functions which includes neurite outgrowth and synapse development, as our data shows [60].

During neurodevelopment, DNA methylation is a key process that regulates the expression of these genes and therefore the maintenance and fate specifications of NPCs [61]. Our results suggest that the premature differentiation observed could be explained by the over-expression of NNAT as a result of the loss of methylation within a CpG island in the promoter observed in the NBS8 organoids. Despite the methylation profile of NBS1 organoids being similar to both control organoids, we cannot entirely exclude the presence of hypomethylation in another regulatory region of the *NNAT* promoter. Hence, further detailed methylation analysis in the NBS organoids should be performed. NNAT is a small transmembrane proteolipid that has been reported to promote neuronal differentiation and a key molecule that maintains cellular homeostasis [62,63]. Interestingly, NNAT expression has been associated with the regulation of stress levels in cells. However, in addition to its protective role, over-expression of NNAT is frequently found in patients with glioblastoma and contributes to the neuronal pathogenesis in Lafora disease, due its strong tendency to mis-fold and aggregate leading to subsequent apoptosis and neuronal loss [63,64].

Day 40 transcriptomic data of NBS organoids revealed an increase in neuron apoptosis along with down-regulation of genes involved in cell division, cell cycle, chromosome segregation and the G2/M damage checkpoint, hence suggestive of a progressive phenotype. With the increase of DNA damage, mimicked here by bleomycin, NBS organoids showed delayed activation of p53 downstream targets and as a result not being able to activate DNA damage checkpoints and stop cell division at the same magnitude seen in control organoids.

Together, our data suggests the *NBN* 657del5 mutation induces a progressive phenotype. In an early developmental stage, cells try to maintain homeostasis and genomic integrity. However, due to the inability to efficiently repair damaged DNA, NPCs undergo premature differentiation with disrupted structural organization. We hypothesize that this process is in part triggered by the over-expression of NNAT. The observed increase in genome instability is a consequence of delayed p53-mediated DNA damage response. Ultimately, the increase in DNA damage together with an aberrant trans-synaptic signaling leads to neuronal apoptosis. We suggest the premature differentiation of the NPCs and an increase in apoptosis in the NPCs and post-mitotic neurons as the mechanisms underlying progressive microcephaly, the hallmark of NBS.

## Data Availability

The datasets generated and analyzed during the current study are available from the corresponding author on reasonable request.

## Supplementary Materials

The following supporting information can be downloaded at: https://www.mdpi.com/article/10.3390/cells11050802/s1, Supplementary Figure S1: Identification of chromosomal aberrations in NBS-iPSCs. Supplementary Figure S2: Gene expression profiles of control and NBS organoids. Supplementary Figure S3: Increase abundance of BIII-Tubulin and DCX-positive neurons in NBS-organoids. Supplementary Figure S4: Analysis of the effect of bleomycin in the down-regulated and exclusively expressed genes in CTR2- and NBS1-organoids. Supplementary Table S1: Overview of the iPSC lines used in this study, Supplementary Table S2: Complete GOs and canonical pathways enrichment analysis of NBS organoids at day 20 as shown in Figure 3 and Figure 5, Supplementary Table S3: Complete GOs and canonical pathways enrichment analysis of CTR2 and NBS1 organoids at day 40 as shown in Figure 6, Supplementary Table S4: Complete GOs and canonical pathways enrichment analysis of the bleomycin treatment in CTR- and NBS organoids at day 40 as shown in Figure 7, Supplementary Table S5: Primer sequences used in this study, Supplementary data 1: Sanger sequencing raw data of *NBN*.
