# Supplementary material for: Impaired p53-Mediated DNA Damage Response Contributes to Microcephaly in Nijmegen Breakage Syndrome Patient-Derived Cerebral Organoids
Source: Cells. 2022 Feb 25;11(5):802. doi: 10.3390/cells11050802 (PMC8909307; doi:10.3390/cells11050802)
Supplement: Supplementary file 1 [file cells-11-00802-s001.zip › Supplementary figures.pdf]

Supplementary Figure S1

A

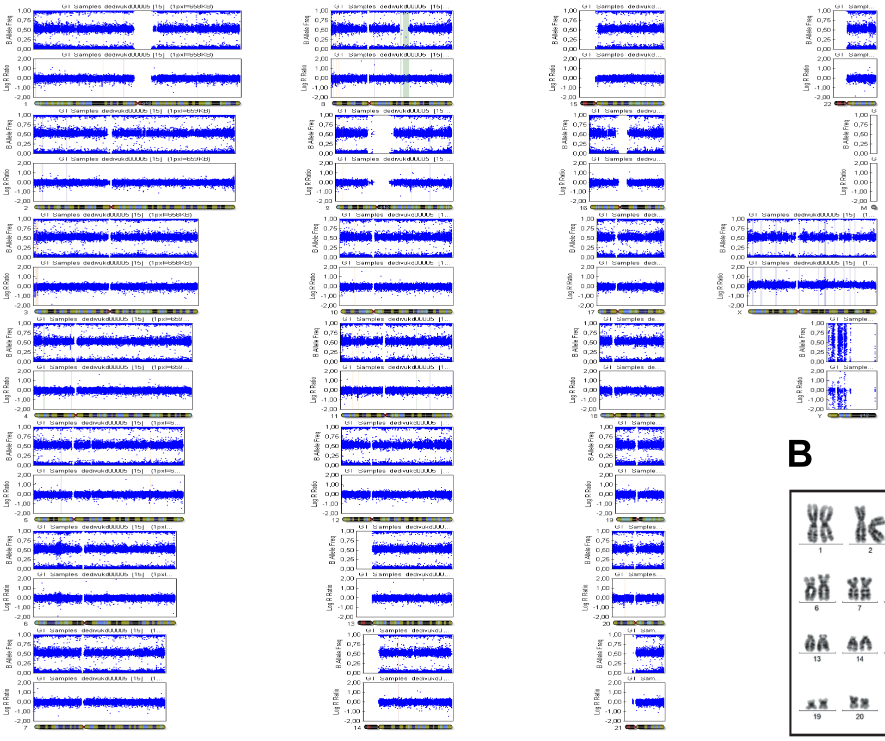

B

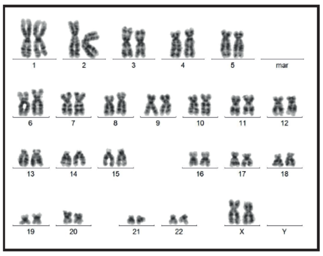

C

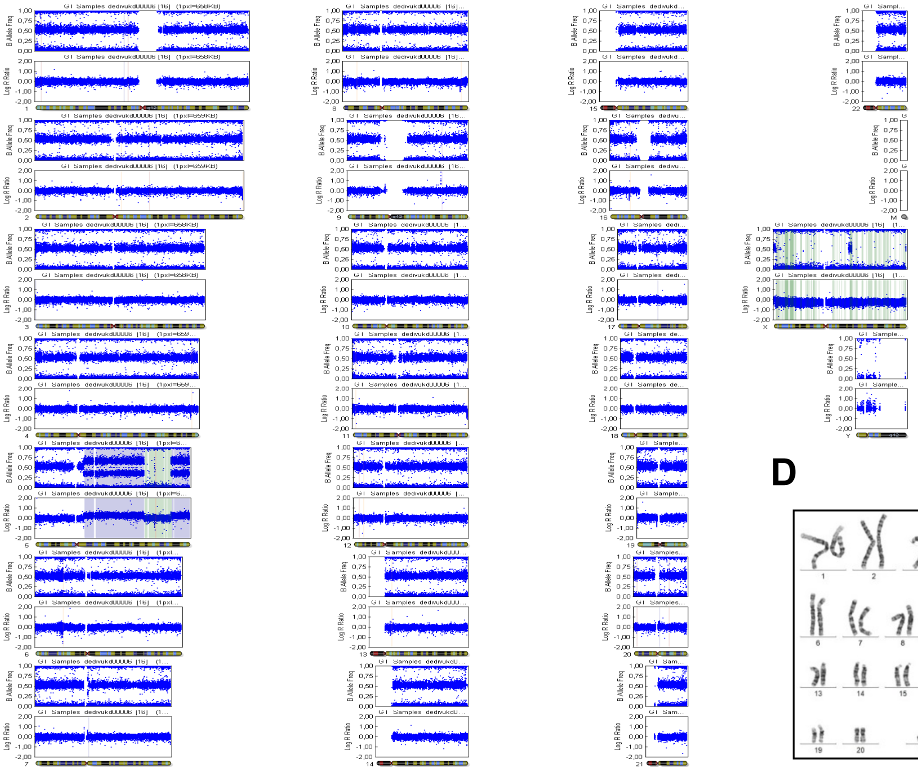

D

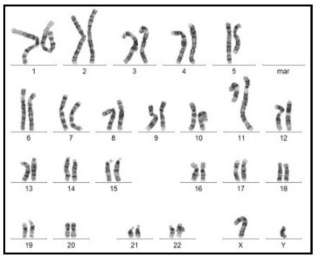

Supplementary Figure S1. Identification of chromosomal aberrations in NBS-iPSCs. (A) Illustration of whole genome profile of CNV analysis after Array-CGH of NBS1-iPSCs showing a LOH in chromosome 8q. CNV, copy number variations; LOH, lost of heterozygosity. (B) G-banding karyotype of NBS1-iPSC: 46,XX. (C) Illustration of whole genome profile of CNV analysis after Array-CGH of NBS8-iPSCs. CNV, copy number variations. (D)G-banding karyotype of NBS8- iPSCs: 46,XY,add(5)(q22),der(11)t(5;11)(q14;q?23)[14]/46,idem,add(4)(q31)[10].

Supplementary Figure S2

A

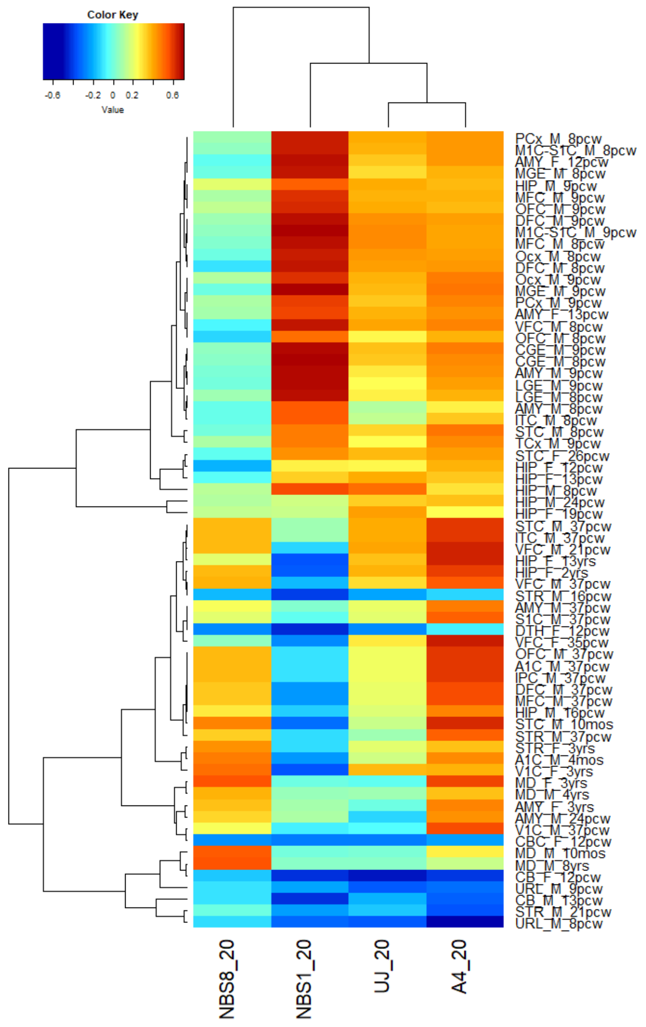

B

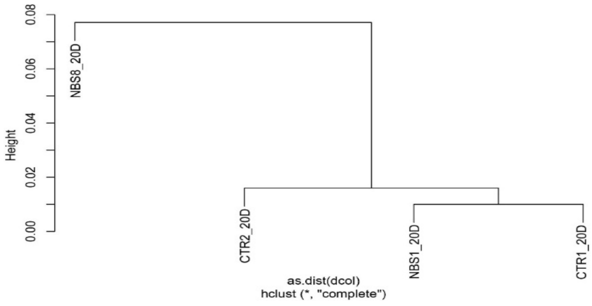

C

**sample**	CTR2_20	NBS1_20	NBS8_20	CTR1_20
CTR2_20	1	0.99241055	0.95085873	0.98756504
NBS1_20	0.99241055	1	0.94271017	0.98813567
NBS8_20	0.95085873	0.94271017	1	0.94954961
CTR1_20	0.98756504	0.98813567	0.94954961	1

Supplementary Figure S2. Gene expression profiles of control and NBS organoids. (A) Heat map of enrichment scores of transcriptomic data from control and NBS organoids in comparison with the Allen Brain Atlas (ABA) human developmental Brain NGS data (https://www.brainspan.org/). (B) Dendogram obtained by hierarchical cluster analysis of microarray-based gene expression data for CTR1, CTR2, NBS1 and NBS8 cerebral organoids. (C) Pearson`s correlation coefficient matrix of the transcriptomic data showing NBS8 has a distinct transcriptome profile.

Supplementary Figure S3

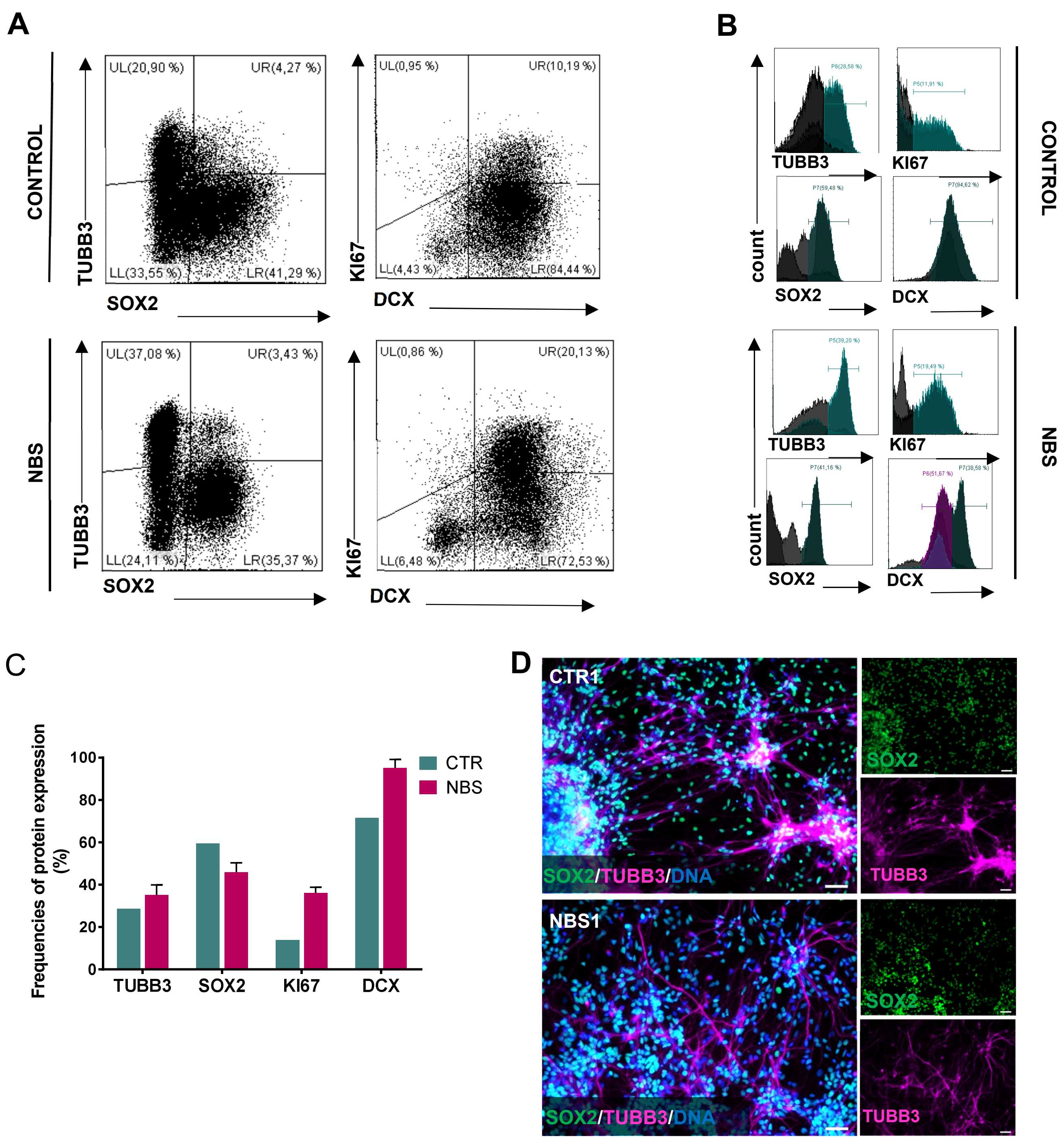

Supplementary Figure S3. Increase abundance of BIII-Tubulin and DCX-positive neurons in NBS-organoids. (A) Expression of BIII-Tubulin (TUBB3), SOX2, KI67 and DCX was assessed in single cell suspension of brain organoids by multicolor flow cytometry . Gates and quadrants were set according to negative controls and frequencies of single- and double-expressors are indicated in these representative dot plots. (B) Representative histograms of single-expression of TUBB3, SOX2, KI67 and DCX in single cell suspension of brain organoids. Gates were set according to negative controls. (C) Quantitative assessment of the protein expression in frequencies (%) Data are representative of n≤ 3 independent experiments and presented as mean ± SD. (D) Representative pictures of immunostainings of SOX2 and βIII-Tubulin in CTR1- and NBS1-organoids after 1 week of single cell dissociation and re-plating in 2D.

Supplementary Figure S4

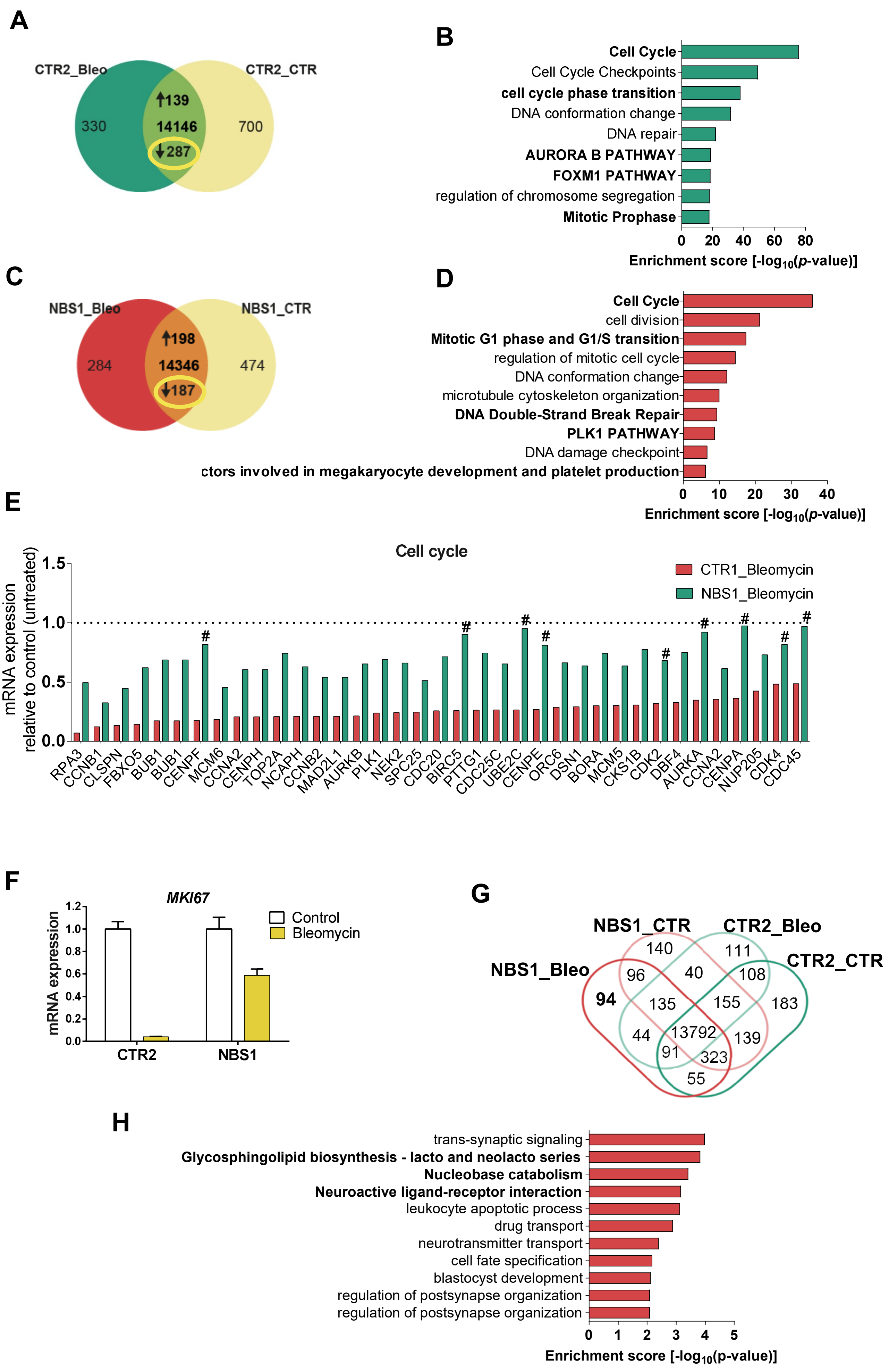

Supplementary Figure S4. Analysis of the effect of bleomycin in the down-regulated and exclusively expressed genes in CTR2- and NBS1-organoids. Relative to Figure 7. (A-B) Venn diagram showing the 287 down-regulated genes (A) in CTR2_Bleomycin-organoids compared to CTR2_control-organoids and the respective bar chart (B) of the enriched clustered GOs and pathways (Top 10 ranked). (C-D) Venn diagram showing the 187 down-regulated genes (A) in NBS1_Bleomycin-organoids compared to NBS1_control-organoids and the respective bar chart (B) of the enriched clustered GOs and pathways (Top 10 ranked). (E) mRNA expression of genes associated with the down-regulated cell cycle pathway in CTR2_Bleomycin-organoids and NBS1_Bleomycin-organoids compared to CTR2_control-organoids and NBS1_control-organoids, respectively. All genes were significantly down-regulated in NBS1 and CTR2 after bleomycin treatment, except genes assigned with # in CTR2_Bleomycin-organoids. (F) qRT-PCR analysis of *MKI67* of mRNA expression in CTR1- and NBS8-organoids after bleomzcin treatment relative to control (untreated) organoids. CTR2: n=3 and NBS1: n=3 technical replicates. Results are mean +/- 95% confidence interval. (G) Venn diagram dissecting 94 exclusively expressed genes expressed in NBS1_Bleomycin-organoids. (H) Bar chart of the enriched clustered GOs and Pathways (Top 10 ranked) 94 exclusively expressed genes in NBS1_Bleomycin-organoids.
