# Supplementary material for: Impaired p53-Mediated DNA Damage Response Contributes to Microcephaly in Nijmegen Breakage Syndrome Patient-Derived Cerebral Organoids
Source: Cells. 2022 Feb 25;11(5):802. doi: 10.3390/cells11050802 (PMC8909307; doi:10.3390/cells11050802)
Supplement: Supplementary file 1 [file cells-11-00802-s001.zip › Supplementary Table S1 and S5.pdf]

Table S1: Overview of the iPSC lines used in this study

IPCS line	Gender	Genotype	Age of the donor	Derived from	Reprogramming method	Karyotype
NBS1	f	Homozygous *NBN* 657del5	18	Human dermal fibroblasts	Episomal reprogramming	46,XX
NBS8	m	Heterozygous *NBN* 657del5	7	Human dermal fibroblasts	Retroviral transduction	46,XX,add(5)(q22), der(11)t(5;11)(q14; q?23)[14]/46,idem, add(4)(q31)[10]
CTR1	m	-		newborn	Retroviral transduction	-
CTR2	m	-		51	Episomal reprogramming	46,XX

Table S5: Primer sequences used in this study

Gene	Forward	Reverse
OTX2	TGCAGGGGTTCTTCTGTGAT	AGGGTCAGAGCAATTGACCA
ATM	AGCTCGGATGCTTTCCTCAA	CTCCATCGAGAAGGTCCACG
CDKN1A	GGAAGACCATGTGGACCTGT	GGCGTTTGGAGTGGTAGAAA
DCX	AAGACTTCAGCCAAGAGCCC	GCGTAGAGATGGGAGACTGC
EMX1	AGACGCAGGTGAAGGTGTGG	CAGGCAGGCAGGCTCTCC
FOXA2	CCACCACCAACCCCACAAAATG	TGCAACACCGTCTCCCCAAAGT
FOXG1	CCCTCCCATITCTGTACGTTT	CTGGCGGCTCTTAGAGAT
HOXA2	TCAGCAAAATGCCCTCTCT	TAGGCCAGCTCCACAGTTCT
HOXB2	TITAGCCGTTCGCTTAGAGG	CGGATAGCTGGAGACAGGAG
HOXB4	ACACCCGCTAACAAATGAGG	GCACGAAAGATGAGGGAGAG
HOXB6	GAACTGAGGAGCGGACTCAC	CTGGGATCAGGGAGTCTTCA
MDM2	AAACTGGGGAGTCTTGAGGG	TGCACATTTGCCTGCTCCTC
MK167	TCGTCCCAGTGGAAGAGTTG	CGACCCCGCTCCTTTTGAT
NNAT	CGGCTGGTACATCTTCCGC	TGTCCCTGGAGGATTTCGAAA
NNAT (bissulfite-specific)	GGGTTAGGTAGTTTGTTGGAA	CCCCCAAACCCTAATAAAT
PAX5	AGGATGCCGCTGATGGAGTAC	TGGAGGAGTGAATCAGCTTGG
SOX2	GAGCTTTGCAGGAAGTTTGC	GCAAGAAGCCTCTCCTTGAA
TP53	CAGGGCAGCTACGGTTTCC	CAGTTGGCAAAACATCTTGTTGAG
